# The VEGFA-Induced MAPK-AKT/PTEN/TGFβ Signal Pathway Enhances Progression and MDR in Gastric Cancer

**DOI:** 10.3390/genes15101266

**Published:** 2024-09-27

**Authors:** Hongming Fang, Yujuan Zhou, Xue Bai, Wanlin Che, Wenxuan Zhang, Danying Zhang, Qingmei Chen, Wei Duan, Guochao Nie, Yingchun Hou

**Affiliations:** 1 College of Life Sciences, Shaanxi Normal University, 620 West Chang-An Street, Xi’an 710119, China; fhm991022@163.com (H.F.); 18326093739@163.com (Y.Z.); bx1620399018@163.com (X.B.); chewanlin0414@163.com (W.C.); 15630431838@163.com (W.Z.); zee06072022@163.com (D.Z.); 2 Guangxi Key Laboratory of Agricultural Resource Chemistry and Biotechnology, 299 Jiao-Yu-Zhong Road, Yulin 537000, China; 18377216921@163.com; 3 School of Medicine, Deakin University, and IMPACT Strategic Research Centre, Melbourne, VIC 3216, Australia; wei.duan@deakin.edu.au

**Keywords:** VEGFA, gastric cancer, MDR, MAPK, PTEN, TGFβ

## Abstract

Background/Objectives: Gastric cancer (GC) is a globally frequent cancer, in particular leading in mortality caused by digestive tract cancers in China. Vascular endothelial growth factor A (VEGFA) is excessively expressed in cancers including GC; its involvement in GC development, particularly in multidrug resistance (MDR), and the signal route it affects in GC remain unknown. To explore the roles VEGFA plays during progression and MDR formation in GC, we studied its function in a VEGFA-deleted GC cell platform. Methods: We initially assessed the importance of VEGFA in GC and MDR using database analysis. Then, using CCK8, wound healing, transwell, scanning electron microscopy, immunofluorescence, flow cytometry, and other techniques, the alterations in tumor malignancy-connected cell behaviors and microstructures were photographed and evaluated in a VEGFA-gene-deleted GC cell line (VEGFA−/−SGC7901). Finally, the mechanism of VEGFA in GC progression and MDR was examined by Western blot. Results: Database analysis revealed a strong correlation between high VEGFA expression and a poor prognosis for GC. The results showed that VEGFA deletion reduced GC cell proliferation and motility and altered microstructures important for motility, such as the depolymerized cytoskeleton. VEGFA deletion inhibited the growth of pseudopodia/filopodia and suppressed the epithelial–mesenchymal transition (EMT). The occurrence of MDR is induced by overactivation of the MAPK-AKT and TGFβ signaling pathways, while PTEN inhibits these pathways. Conclusions: All findings suggested that VEGFA acts as a cancer enhancer and MDR inducer in GC via the MAPK-AKT/PTEN/TGFβ signal pathway.

## 1. Introduction

The family of vascular endothelial growth factor (VEGF) is crucial for angiogenesis and was identified 25 years ago [1]. VEGF has multiple effects on many cell behaviors, especially on endothelial cells for their homeostasis and relevant diseases [2,3]. VEGF has been found to be a major molecular etiology of heightened pathology in various blinding eye diseases, including cancer growth and metastasis, while being crucial for maintaining vascular homeostasis in a range of cells and tissues, notably during embryonic development [4,5].

At present, the incidence of gastric cancer (GC) is the fourth-highest worldwide, with more than 1 million people diagnosed each year [6,7,8], accounting for almost 800,000 fatalities per year due to its poor early diagnosis rate and high mortality rate [9,10]. The incidence of GC is much higher in Asia, particularly in China, than in North America [6,7,8]. It should be emphasized here that the accumulation of mutations in numerous varied genes over time causes GC oncogenesis [11]. However, the roles that VEGFA performs in the progression of GC remain poorly unknown.

Multidrug resistance (MDR) is one of the core reasons that cause poor prognosis in many cancer patients, including those with GC [11,12], but the roles VEGF plays in GC MDR induction are still unclear.

Based on our previous bioinformatical assay for VEGF family expression in GC, we found that the expression level of vascular endothelial growth factor A (VEGFA) is higher than vascular endothelial growth factor B (VEGFB) [11]. To explore the functions VEGFA plays during GC development, we deleted VEGFA expression in a GC cell line using the CRISPR/Cas9n technology, then a series of molecular and cellular biological experiments were conducted to validate the roles played by VEGFA via signal regulation.

## 2. Materials and Methods

### 2.1. Cell Culture

Human embryonic kidney cells HEK-293 and human GC cells SGC7901 were obtained from the ATCC bank of the Chinese Academy of Sciences (Shanghai, China). SGC7901 cells were cultured with RPMI1640 medium, and HEK-293 cells were cultured with DMEM medium. Then, 10% fetal bovine serum (FBS; Gibco, Gaithersburg, MA, USA), 100 µg/mL streptomycin (Sigma, St. Louis, MO, USA), and 100 U/mL penicillin (Sigma, St. Louis, MO, USA) were added to both cultures. At 37 °C, cells were grown in a humid environment (5% CO_2_).

### 2.2. Obtainment of VEGFA Knockout GC Cell Line

The SGC7901 cell line with the VEGFA gene removed (VEGFA-/-SGC7901) was created using CRISPR/Cas9n. VEGFA sgRNA-a (5′-CACCGAAGATGTACTCGATCTCATC-3′) and VEGFA sgRNA-b (5′-CACCGTGCCCCTGATGCGATGCGG-3′), which target exon 3 of VEGFA, were created by the authors and then inserted into the PX462 V2.0 vectors purchased from AddGene (Watertown, NA, USA). Using Lipofectamine 2000 (Invitrogen, Carlsbad, CA, USA), the aforementioned vectors, tagged with green fluorescent protein (GFP), were transfected into SGC7901 cells. The intensity of cell fluorescence was used to assess transfection effectiveness. The transfected cells were then exposed to 0.25 µg/mL puromycin antibiotic selection 24 h after transfection, and the puromycin-selected cells were then monoclonalized in used media. Finally, positive clone cells were discovered by sequencing and confirmed using Western blot and immunohistochemistry. The VEGFA with two alleles deleted was designated VEGFA−/−SGC7901, while the VEGFA with one allele removed was designated VEGFA+/−SGC7901.

### 2.3. Real-Time PCR

A RNeasy kit (Qiagen, Germany) was used to extract total RNA. The primers of VEGF were the following: 5′-ATGGCAGAAGGAGGAGGG-3′ (forward); 5′-TGGCCTTGGTGAGGTTTG-3′ (reverse). The primers of U6 were the following: 5′-AGAGAAGATTAGCATGGCCCCTG-3′ (forward); 5′-AGTGCAGGGTCCGAGGTATT-3′ (reverse). The primers of GAPDH were the following: 5′-AATCCCATCACCATCTTCCA-3′ (forward); 5′-CCTGCTTCACCACCTTCTTG-3′ (reverse).

### 2.4. Cell Proliferation Assay

For the CCK8 assay, we used Cell Counting Kit-8 (MedChem Express, US), and the ELX800 ELISA reader (Bio-Tek, Winooski, VT, USA) was used to read the absorbance value (OD) at 450 nm. Six-well plates with 0.5 × 10^3^ cells planted in each well were used for the colony formation test. At 37 °C, cells were grown in a humid environment (5% CO_2_). Cells were stained with 0.1% crystal violet (Sigma, St. Louis, MO, USA) after two weeks of incubation, fixed with 4% paraformaldehyde, and counted by a hand counter.

### 2.5. Cell Motility Assay

When the density of cells met the experimental standard for the wound healing experiment, the layer of cultivated cells was scraped with a point. Photographs were taken under a normal microscope (Sunlight Optical Technology, Zhongshan, China) at 0, 24, 48, and 72 h. Image J was used to analyze and calculate the migration rate of each group. For the transwell assay, the upper chamber (JET Biofil Biotech, Guangzhou, China) was seeded with approximately 8 × 10^3^ cells per well and starved for culture, while the lower chamber was supplemented with RPMI1640 medium containing 20% FBS. After 24 h of conventional culture, the cells were treated with methanol, stained with 0.1% crystal violet, and imaged under a microscope. Finally, using an ELX800 ELISA reader, the absorbance value was measured at 562 nm in order to reflect migration cell count.

### 2.6. Flow Cytometry Assay (FCM)

Approximately 1 × 10^6^ cells were seeded in each cell culture dish. Cells were starved for 24 h, treated with 70% ethanol for 12 h at 4 °C, and then stained with 0.05 mg/mL propidium iodide (PI) containing RNase A for 30 min. The cells were then subjected to a FCM (Beckman-Coulter, Brea, CA, USA) analysis.

### 2.7. Scanning Electron Microscopy (SEM)

Approximately 1 × 10^6^ cells were seeded in each cell culture dish with a small cover-glass slip in each dish. The slips were dehydrated in gradient diluted ethanol after fixation in 2.5% glutaraldehyde (Sigma, St. Louis, MO, USA) for 24 h, then the cells were then dehydrated at 4 °C overnight. Finally, a SEM (Quanta 200, Hillsboro, OR, USA) was utilized to observe the cells.

### 2.8. Immunocytochemistry/Fluorescence Assays

The cell seeding and grouping were same as above. The synchronized cultivated cells on a small cover-glass slip were treated with 4% paraformaldehyde for 15 min, transparentized in 0.3% Triton X-100 for 5 min, and blocked in 1% BSA blocking solution for 45 min. For immunocytochemical assays, rabbit anti-human antibodies VEGFA (1:100), β-tubulin (1:200), Vimentin (1:50), and Lamin-B (1:500) were incubated at 4 °C for 12 h. After that, the cells were incubated with HRP-labeled goat anti-rabbit IgG antibody for one hour at 37 °C (1:50). The above antibodies were purchased from Sangon Biotechnology Co., Ltd. (Shanghai, China). The images were imaged under a light microscope (Sunlight Optical Technology, Zhongshan, China). For immunofluorescence assays, cells were exposed to rhodamine-phalloidin F-actin (1:500; Thermo Fisher Scientific, Waltham, MA, USA) for 1 h. Nuclei were visualized by DAPI (4′6-diamine-2-phenylindole) staining. Apoptosis was studied using the Annexin V-FITC Apoptosis Detection Kit (Beyotime Biotechnology, Shanghai, China). The mixture was kept at an ambient temperature and shielded from light for 15 min. Cells were observed with an inverted fluorescent microscope (DMIL LED, Leica, Germany) and fluorescence intensity was determined using Image J (version 1.53).

### 2.9. Western Blot Assay

The Protein Extraction Reagent Kit (Sigma-Aldrich, St. Louis, MO, USA) was used to extract cellular proteins. Equal quantities of protein samples were added to the corresponding gel holes, followed by electrically applied glue running and protein transfer to NC membranes (Whatman, Medstone, Kent, UK), which were incubated at 4 °C for 12 h with rabbit-anti human VEGFA (1:500), MMP-9 (1:1000), SNAI1 (1:500), VIM (1:2000), CDHE (1:500), P-gp (1:200), ABCC5 (1:950), EZH2 (1:300), STX1A (1:500), COX-2 (1:500), MEK1/2 (1:1000), AKT (1:1000), FOS (1:500), SMAD2 (1:300), RAS (1:200), PTEN (1:500), and GAPDH (1:1600) antibodies, followed by 1.5 h incubation with the HRP-labeled goat anti-rabbit IgG (1:8000) at normal atmospheric temperature. The above antibodies were purchased from Sangon Biotechnology Co., Ltd. (Shanghai, China). Proteins on the NC membrane were developed using ECL kits (Vazyme Biotech, Nanjing, China) and data analysis was performed.

### 2.10. Statistical Analysis

Statistical analyses were performed using Prism 7 (GraphPad, Boston, MA, USA). Unless otherwise specified, each experiment was performed with at least three replicates. Student’s *t* test was used for two-group comparisons, and one-way ANOVA was used for multigroup comparisons. *p* < 0.05 was considered statistically significant. (* *p* < 0.05, ** *p* < 0.01, *** *p* < 0.001, **** *p* < 0.0001, “ns” indicates no significant difference).

## 3. Results

### 3.1. The Prognosis of GC Patients Is Positively Associated with VEGFA Expression

**Figure 1:**
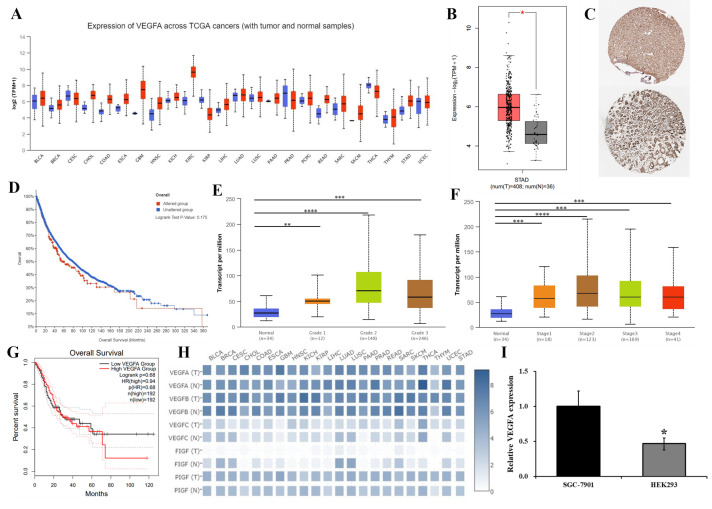
Different databases were evaluated to determine the effect of VEGFA on GC progression. (**A**,**B**) VEGFA expression in various cancers (**A**) and in GC (**B**). (**C**) The HPA database immunohistochemical samples (pictured above is normal tissue; below is GC tissue). (**D**) The relationship between VEGFA mutation and prognosis in GC patients. (**E**,**F**) VEGFA in different differentiation degree and pathological staging in GC. (**G**) Survival curve analysis of VEGFA in GC patients (TCGA). (**H**) VEGF family expression in various cancers. (**I**) The expression of VEGFA in SGC7901 cells was detected by RT-PCR. * *p* < 0.05, ** *p* < 0.01, *** *p* < 0.001, **** *p* <0.0001.

Databases such as GEPIA, UALCAN, and Human Protein Atlas (HPA) were used to test the relationship between VEGFA expression and GC development. For more information, see http://gepia.cancer-pku.cn (accessed on 1 June 2021), http://ualcan.path.uab.edu/ (accessed on 1 June 2021), and http://www.proteinatlas.org (accessed on 1 June 2021). The findings revealed that VEGFA expression is consistently abundantly higher in GC when compared to 408 normal gastric tissues and 36 GC tissues (Figure 1A,B), and it is substantially raised in pathological tissue section of GC (Figure 1C). According to the prognostic significance assessment of the VEGFA mutation, the prognosis for GC patients who had these mutations may be poorer in different patients with different mutations of some signal pathways (Figure 1D). The study of VEGFA expression in GC samples with various clinical stages and tumor grades showed that patients had a worse prognosis and that less differentiation of GC was followed by a greater level of VEGFA expression, particularly in the later stages of GC (Figure 1E–H). The endogenous VEGFA expression in SGC7901 cells was found and displayed in Figure 1I to confirm the aforementioned prediction. According to the data above, higher VEGFA expression and mutation are strongly positively linked with a bad prognosis for GC.

### 3.2. Establishment of VEGFA-Knockout SGC7901 Cell Line via CRISPR/Cas9n

**Figure 2:**
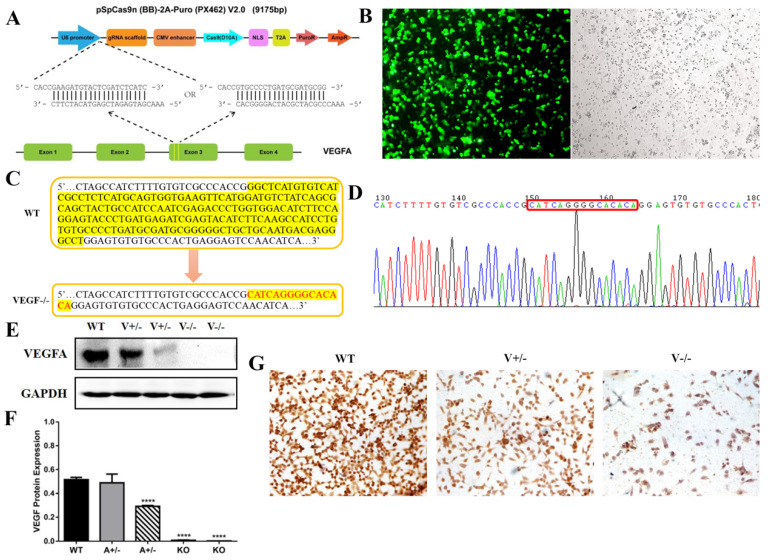
VEGFA-deleted SGC7901 cell line was generated with CRISPR/Cas9n. (**A**) The designation for VEGFA deletion. (**B**) The efficiency (>80%) of the transfection of CRISPR/Cas9n vector at 60 h post-transfection (4×). (**C**,**D**) Sequencing assay for positive clone selection. (**E**–**G**) The validation of the VEGFA-deleted clone by Western blot ((**E**,**F**), **** *p* < 0.0001) and immunocytochemistry assay ((**G**), 20×).

To validate the roles VEGFA played in progression of GC objectively, CRISPR/Cas9n was used to form VEGFA deletion in SGC7901 cells. We coupled sgRNAs (sgRNA-a, sgRNA-b) targeting exon 3 of VEGFA (Figure 2A), and the vectors were named CRISPR/Cas9n-VEGF-a and CRISPR/Cas9n-VEGF-b. At 60 h after the transfection (Lipofectamine 2000, GIBCO, Grand Island, NY, US), the SGC7901 cells’ transfection efficiency was greater than 80% (Figure 2B). By the genome sequences alignment with wild type SGC7901 cells, the positive cell clone was mutated, with the 185bp fragment at the target site being replaced by a 15bp fragment (CATCAGGGGCACACA) (Figure 2C,D). The clone of VEGFA−/−SGC7901 was confirmed by the results of Western blot and immunocytochemistry assay (Figure 2E–G).

### 3.3. Deletion of VEGFA Suppressed the Growth and Mobility and Remodeled Microstructure in GC Cells

The malignancy of cancer cells is dependent on their growth, invasion, and metastasis, but all are correlated with enhanced polymerization of cytoskeletons [13,14,15]. In our case, the malignancy changes of SGC7901 cells caused by VEGFA deletion were explored with a series of detections as follows.

**Figure 3:**
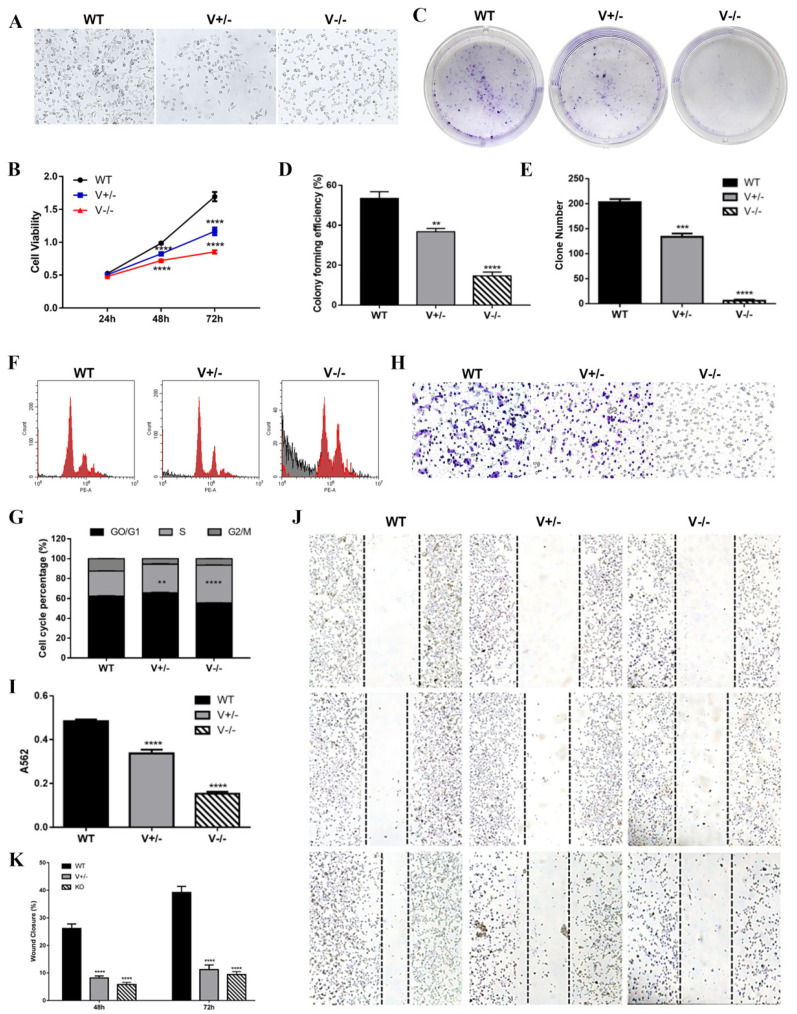
VEGFA deletion reduced the multiplication and viability of SGC7901 cells. (**A**) Cell morphology of each group under light microscope (10×). (**B**,**C**) Results of CCK8 (**B**) and colony formation (**C**). (**G**,**H**) Digital assays for colony formatting efficiency (**G**) and clone counting (**H**). (**D**,**I**) Cell cycle analysis by FCM (**D**) and data assay (**I**). (**E**,**J**) Results of transwell. (**F**,**K**) Results of wound healing. (** *p* < 0.01, *** *p* < 0.001, **** *p* < 0.0001).

VEGFA deletion caused SGC7901 cells contact inhibition to be rescued (Figure 3A), proliferation was inhibited significantly (Figure 3B–E), and the cell cycle was stopped at the S phase (Figure 3F,G). When VEGFA was eliminated, SGC7901 cells’ deformability (Figure 3H,I) and migration (Figure 3J,K) were noticeably decreased.

**Figure 4:**
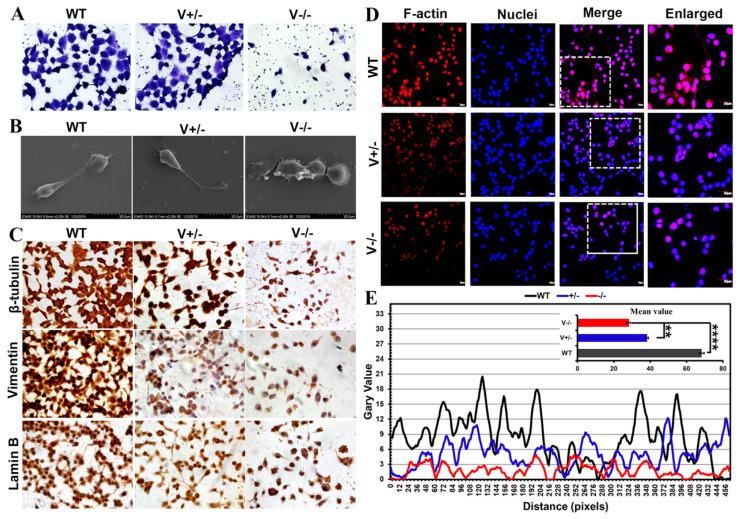
VEGFA deletion caused cyto/nucleoskeletal remodeling. (**A**,**B**) Results of Coomassie Brilliant Blue staining (40×) under optical microscope (**A**) and SEM (**B**) (scale bar: 20 μm). (**C**–**E**) The cyto/nucleoskeletal microstructures displayed by immunocytochemistry (**C**) and immunofluorescence assays ((**D**), 40×, scale bar: 20 μm), and data assay for F-actin ((**E**), ** *p* < 0.01, **** *p* <0.0001).

VEGFA deletion caused microstructure development to be inhibited and contact inhibition to be rescued (Figure 4A,B). As the elements of the cyto/nucleoskeleton, β-tubulin, vimentin, lamin B (Figure 4C), and F-actin (Figure 4D,E) were seen to be disrupted and depolymerized following VEGFA deletion. Together, these findings demonstrated that VEGFA affects cyto/nucleoskeletal microstructure formation, and therefore, as a key factor, VEGFA plays role in enhancing the malignancy in GC.

### 3.4. VEGFA Deletion Modulated Apoptosis in SGC7901 Cells

**Figure 5:**
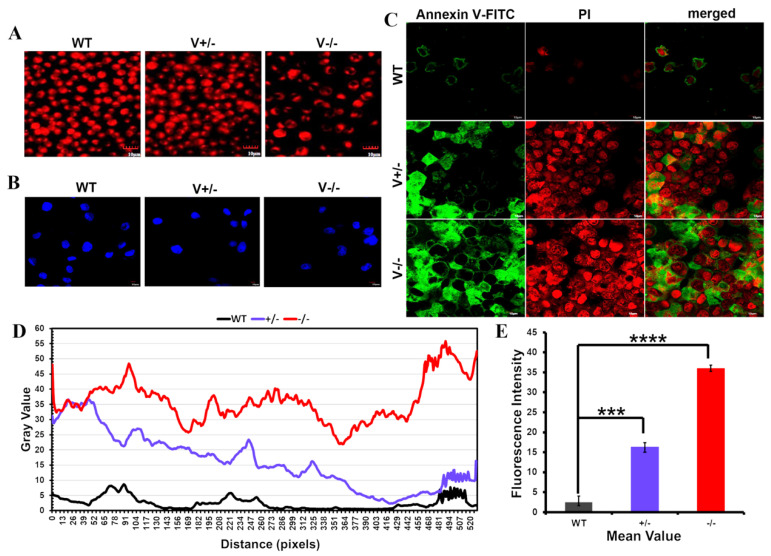
The tendency of apoptosis in SGC7901 cells induced by VEGFA deletion. (**A**) MitoScene 633 staining and (**B**) DAPI staining (scale bar: 10 μm). (**C**–**E**) Apoptosis detection by annexin V FITC antibody and PI under a fluorescence microscope ((**C**), scale bar: 10 μm). (*** *p* < 0.001, **** *p* < 0.0001).

In VEGFA-deleted cells, the mitochondrial membrane potential was decreased (Figure 5A), chromatin was highly concentrated, and nuclear pyknosis or nucleolysis was observed (Figure 5B). Notably, at the end of apoptosis, Annexin V bound to Phosphatidylserine (PS), and PI entered the inner cell membrane and bound to DNA (Figure 5C–E). According to the data above, SGC7901 cells tended to undergo apoptosis when VEGFA expression was reduced.

### 3.5. VEGFA Serves as an Enhancer for GC and MDR via MAPK-AKT/PTEN/TGFβ Pathways

**Figure 6:**
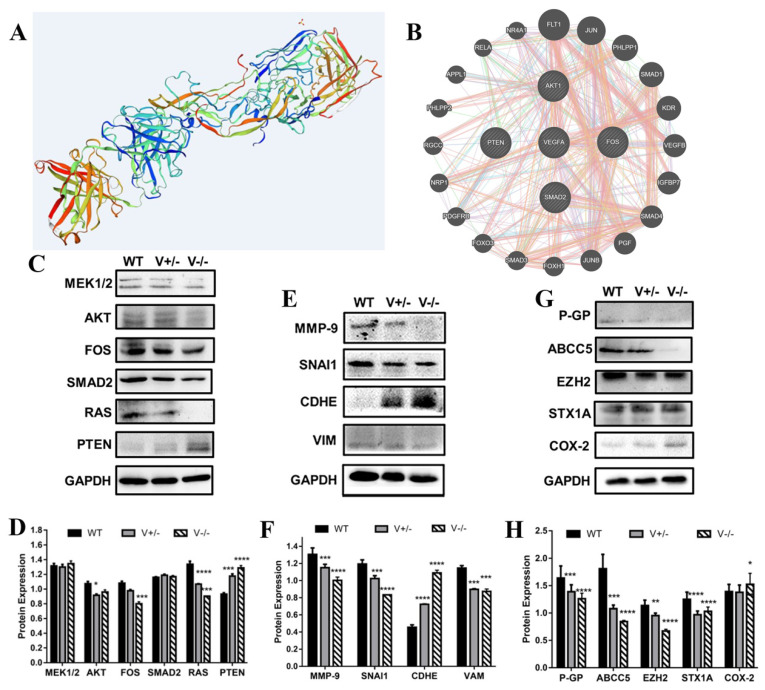
The mechanism VEGFA utilizes to enhance oncogenesis, progression, and MDR in GC. (**A**) Simulated spatial configuration of VEGFA (SWISS-MODEL database). (**B**) Prediction of interactions between VEGFA and other genes (STRING database). (**C**–**H**) Detection of the expression of the key signal genes (**C**,D), EMT-relevant genes (**E**,**F**), and MDR-relevant genes (**G**,**H**) in SGC7901 cells. (* *p* < 0.05, ** *p* < 0.01, *** *p* < 0.001, **** *p* < 0.0001).

The SWISS-MODEL assay (https://swissmodel.expasy.org, (accessed on 1 June 2021)) revealed that the structure of VEGFA, in addition to the classic protein secondary structure, also contains multiple ligand binding sites (Figure 6A). The STRING database (https://string-db.org, (accessed on 1 June 2021)) predicted that VEGFA interacts with some genes including AKT (protein kinase B), RAS (ras protein), MEK1/2 (MAP kinase/ERK kinase 1/2), FOS (fos proto-oncogene), SMAD2 (SMAD family member 2), and PTEN (anti-oncogene), which implies that the pathways of MAPK (mitogen-activated protein kinase)-AKT (protein kinase B) and TGFβ (transforming growth factor-β) are those that the VEGFA signal merges with (Figure 6B).

VEGFA deletion resulted in significant inhibition of the expression of other key genes mentioned above, except PTEN expression in GC cells. As a suppressor of some cancer-enhancing signals [16], PTEN expression is negatively correlated with VEGFA expression. These findings suggest that VEGFA contributes to GC progression through MAPK-AKT/PTEN/TGFβ signaling pathways (Figure 6C,D).

The main EMT (epithelial-mesenchymal transition) markers include SNAI1 (snail family transcriptional repressor 1), MMP-9 (matrix metallopeptidase 9), CDHE (cadherin 1), and VIM (vimentin) [17,18]. When VEGFA was knocked out, the expression of MMP-9, SNAI1, and VIM was reduced while that of CDHE increased (Figure 6E,F), indicating that VEGFA promotes EMT in GC.

MDR (multidrug resistance) is an important obstacle for cancer chemotherapy. P-gp (P-glyco-protein, encoded by ABCB1), ABCC5 (ATP binding cassette subfamily C member 5), EZH2 (enhancer of zeste homolog 2), STX1A (syntaxin 1A), and COX-2 (cyclooxygenase-2) serve as MDR enhancers that lead to the poor prognosis of cancer chemotherapeutics, according to our previously published data [19,20,21]. The results (Figure 6G,H) demonstrated that, except for COX-2, the deletion of VEGFA resulted in much lower expression of P-gp, ABCC5, EZH2, and STX1A. These results suggest that VEGF is a MDR enhancer in GC, but for COX-2, its interaction with VEGFA and role in MDR in GC remain open to further study.

## 4. Discussion

According to recent research, the VEGF gene also affects the tumor microenvironment in addition to its effects on angiogenesis and vascular permeability [22,23,24,25]. Additionally, VEGF receptors could control how fibroblasts in the stroma of a tumor operate [26,27,28]. According to the early studies that examined how VEGF affected tumor cells, non-ribosomal peptide synthetases (NRPs) and receptor protein tyrosine kinases (RTKs) are VEGF-mediated proteins that can encourage cancer cell multiplication, survival, migration, and invasion [29,30,31,32,33]. Studies have suggested that VEGF regulates these processes through well-known signaling pathways such as the PI3K-AKT and MAPK pathways [34]. VEGFA may therefore be thought of as a possible target for tumor diagnostics and treatment.

GC, defined as a cancer caused by the accumulation of multiple important gene mutations, is a global public health problem [35,36]. The absence of identifiable molecular targets and adequate countermeasures is the primary cause for GC’s low therapeutic effect. Therefore, discovering new molecular treatment targets for GC is critical. VEGFA is implicated in cancer angiogenesis, tumor development, and metastasis [3,37]. However, the functions of VEGFA during GC growth must still be objectively confirmed using repeatable experimental conditions.

We attempted to investigate the functions of VEGFA in GC progression in the absence of VEGFA using the CRISPR/Cas9n technique. The oncogenic role of VEGFA in GC advancement was anticipated and determined using tests from various databases, as shown in Figure 1, and VEGFA performs essential roles in GC as a cancer promoter. The VEGFA−/−SGC7901 cell line was used to confirm the prediction. In further investigations, it was shown that VEGFA significantly boosted GC malignancy by promoting SGC7901 cellular proliferation, migration, and motility as well as remodeling cellular motility-related microstructures. Indicators of malignancy include the dramatic reconfiguration of cytoskeletal networks and the increased polymerization of actin/tubulin contractility that follow its modulation to preserve cell microstructures. These changes contribute to cancer cell invasion and spread [38]. Our findings showed that the deletion of VEGFA significantly reduced cell motility, markedly prevented the growth of pseudopodia/filopodia, and simultaneously stimulated the restoration of contact inhibition.

Following VEGFA deletion in GC cells, the EMT process is reversed and an MET (mesenchymal–epithelial transition) tendency is displayed, and these alterations cause the malignancy to be blocked and chemotherapy sensitization in GC. In order to investigate the routes via which VEGFA increases MDR development, the expression of genes important to MDR was investigated. The findings showed that deletion of VEGFA resulted in increased expression of PTEN, an inhibitor of pathways that promote cancer, as well as decreased expression of the members (MEK1/2 and AKT) of MAPK and the member (SMAD2) of TGFβ signaling pathways [16]. As a critical mechanism, the MAPK signal pathway controls cell proliferation, migration, dedifferentiation, EMT, and metastasis in a variety of malignancies [39]. Given that PTEN regulates the AKT-mediated TGFβ/SMAD2 signaling pathway negatively, loss of VEGFA may block PTEN degradation, which eventually results in the deactivation of this signaling pathway [40,41,42].

**Figure 7:**
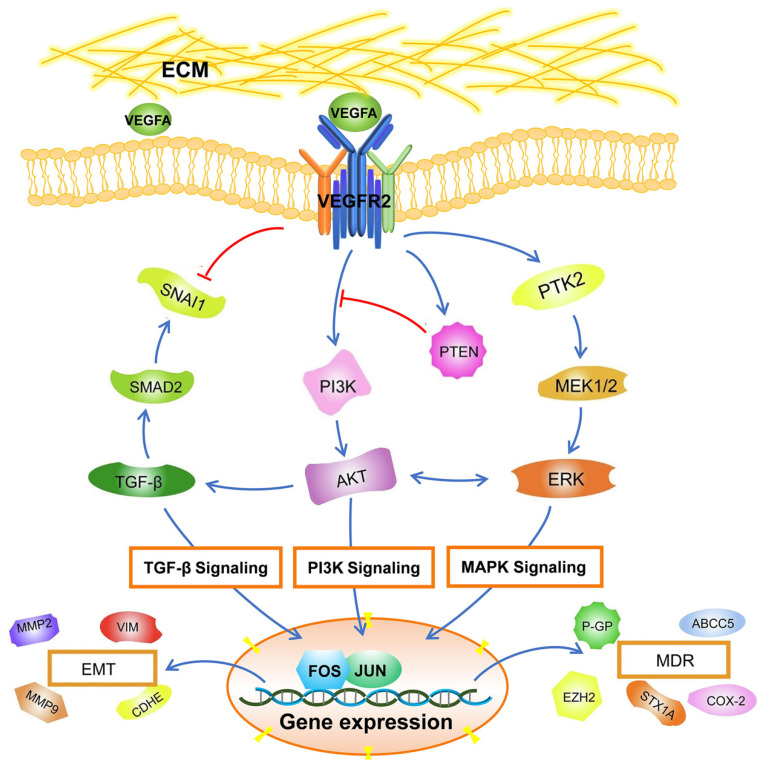
An illustration of the VEGFA regulatory mechanism in GC.

Figure 7 depicts a schematic signaling illustration of how VEGFA acts in GC advancement based on our results and reported data.

## 5. Conclusions

Taken together, our findings show that VEGFA accelerates GC oncogenesis and progression via the MAPK-AKT/PTEN/TGFβ signaling pathways, and VEGFA has potential to be evaluated as a diagnostic marker for GC diagnosis and target for GC chemotherapy. The pathway and its inducer VEGFA will need to be further validated in GC cells and animal models. The advances of this study will benefit GC clinical practice and diagnostic paradigms as a reference, and more importantly, it may provide a reference to benefit the designation and development of GC-targeting chemotherapeutic drug formulation as in our previous report [12].

## Data Availability

The datasets generated during and analyzed during the current study are available from the corresponding author on reasonable request. The data are not publicly available due to privacy restrictions.
